# Relationship between thyroid function and lipid atherogenic profile in pediatric patients with multisystem inflammatory syndrome associated with COVID-19

**DOI:** 10.3389/fped.2024.1456545

**Published:** 2024-10-29

**Authors:** Valeria Calcaterra, Raffaella De Santis, Davide Braghieri, Sara Zanelli, Gianvincenzo Zuccotti

**Affiliations:** ^1^ Department of Internal Medicine and Therapeutics, University of Pavia, Pavia, Italy; ^2^ Pediatric Department, “Vittore Buzzi” Children’s Hospital, Milano, Italy; ^3^ Department of Biomedical and Clinical Science, University of Milano, Milano, Italy

**Keywords:** thyroid, non-thyroidal illness syndrome, multisystem inflammatory syndrome, children thyroid hormones, lipid metabolism, lipid atherogenic profile

## Abstract

**Introduction:**

Concurrent alterations in the metabolic profile and thyroid dysfunction, including non-thyroidal illness syndrome (NTIS) has been reported in multisystem inflammatory syndrome in children (MIS-C). Considering the influence of thyroid hormones (TH) on lipid metabolism, we explored the relationship between thyroid function and the atherogenic lipid profile in children with MIS-C at admission and during a 12-month follow-up.

**Patients and methods:**

we considered children admitted for MIS-C. Total and HDL cholesterol, triglycerides (TG), fasting plasma glucose, fasting plasma insulin as well as free T3 (FT3), free T4 (FT4), and TSH were assessed at diagnosis within 24 h of admission and during follow-up. TG/HDL ratio, no-HDL/HDL ratio and atherogenic index of plasma was also considered as atherogenic risk markers.

**Results:**

we monitored 56 children. On admission, pathological levels of FT3, FT4, TSH, TG, TC, HDL, TG/HDL ratio, no-HDL/HDL ratio, and AIP were detected. Correlation analyses revealed associations between FT3, FT4, and lipid markers and TSH with TG. During monitoring, while complete restoration of TH balance was achieved at 12 months, some patients still exhibited an altered lipid profile, without correlation between thyroid function and lipid markers.

**Conclusions:**

we supported a relationship between thyroid function and an atherogenic lipid profile in children with MIS-C. This may result from interactions between adaptive and innate metabolic responses and genetic predisposition. Elucidating the relationship between TH and metabolic pathways during infections could help identify new biomarkers to prevent acute and fatal outcomes, improving patient prognosis and protecting long-term health.

## 1 Introduction

Multisystem inflammatory syndrome in children (MIS-C) is a post-infectious condition that occurs after a COVID-19 infection (1–3). According to the Centers for Disease Control and Prevention (CDC) criteria (4), MIS-C affects individuals under 21 who have recently tested positive for SARS-CoV-2 or had COVID-19 exposure within the preceding four weeks. The predominant theory suggests that post-infectious hyper-inflammation and cytokine storms contribute to the pathophysiology of MIS-C (5, 6). Literature indicates that the clinical spectrum of COVID-19 varies due to diverse immune responses to the virus, potentially leading to inflammatory exacerbations that affect the heart, lungs, kidneys, brain, skin, eyes, gastrointestinal organs, and endocrine system (1–3, 7).

Regarding the involvement of endocrine organs, several reports have described the association between SARS-CoV-2 infection and various thyroid diseases, including non-thyroidal illness syndrome (NTIS) defined as abnormal findings on thyroid function tests that occur in the context of a non-thyroidal illness, without preexisting dysfunction of the hypothalamic-pituitary-thyroid axis, thyrotoxicosis, hypothyroidism, isolated elevated free T4, and isolated low free T4 (8).

Specifically, Ashrafi et al. conducted a systematic review and meta-analysis and found a 26% prevalence of NTIS (8). The relationship between COVID-19 and NTIS has also been observed in children and adolescents (9, 10). We previously observed that more than 90% of children with SARS-CoV-2-associated MIS-C exhibited NTIS (10), which resolved during follow-up (11, 12).

NTIS is common among critically ill children and appears to be associated with mortality and illness severity. Carreras et al. (13) reported that NTIS is linked to adverse outcomes in these children, with FT4 and FT3 values at admission potentially serving as good predictors of high mortality risk. NTIS has been interpreted as evidence of an adaptation to preserve energy during critical illness and hypercatabolism (10, 11). This hypothesis has also been suggested in patients with MIS-C, supported by concurrent alterations in the metabolic profile, such as a high prevalence of insulin resistance (IR), glycemic fluctuation, and dyslipidemia (10, 14).

Studies in adults have found that COVID-19 infection is associated with altered lipid levels and cardiovascular disease. Specifically, high values of the atherogenic index are associated with prolonged hospital stays, poor prognosis, and increased disease severity (15).

Considering the influence of thyroid hormones (TH) on lipid metabolism (16, 17), our study aimed to explore the relationship between thyroid function and the atherogenic lipid profile in children with MIS-C, both at admission and during a 12-month follow-up period. Improving the understanding of the relationship between thyroid function and lipid profile during infections may help identify new biomarkers to prevent poor prognosis.

## 2 Patients and methods

### 2.1 Patients

We included in the study children admitted to the Pediatric Department of the Buzzi Children’s Hospital (Milano, Italy), between November 2020 and July 2022, for MIS-C according to the CDC classification (4). Specifically, the inclusion criteria were: fever (≥38°C); clinical severity requiring hospitalization; evidence of systemic inflammation (CRP ≥ 3.0 mg/dl or 30 mg/L); new onset symptoms in at least two of the following categories: cardiac involvement, mucocutaneous involvement, shock, gastrointestinal symptoms, or hematologic involvement (platelet count <150,000 cells/µl, or absolute lymphocyte count <1000 cells/µl); and laboratory confirmation of SARS-CoV-2 infection (through RNA detection, specific antigen detection within 60 days prior to or during hospitalization, or detection of SARS-CoV-2-specific antibodies) (4).

Children with pre-existing thyroid disease and/or lipid disorders were excluded.

To define the severity of multisystemic involvement, we used a score previously proposed by our group that considers the severity scores as the sum of each of the sub-scores, including damage to each organ and system (kidney, heart, gastrointestinal system, central nervous system, lung, skin/mucosal, endocrine and metabolic, weight loss, electrolyte imbalance), duration of fever, duration of hospitalization in the pediatric intensive care unit, duration of hospitalization (11).

Auxological findings, biochemical and hormonal profiles were recorded at diagnosis and two subsequent follow-up visits, at six- and twelve-months post-diagnosis.

The study complied with the Declaration of Helsinki and received approval from the institutional ethics committee (MI-1, n. 0034170; protocol number 2021/ST/138). After explaining the study's nature, written consent was obtained from the guardians.

### 2.2 Auxological parameters

Physical examinations of children with MIS-C included measurements of weight, height, BMI, and pubertal stage based on Marshall and Tanner criteria (18, 19).

Height was measured while the patient stood barefoot using a wall-mounted Harpenden stadiometer, with an approximate precision of ±1 mm. Weight was measured with the subjects wearing only underwear, standing upright on a platform scale with an approximate precision of ±100 g (20, 21).

BMI was calculated as body weight in kilograms divided by height in meters squared and was transformed into BMI z scores using WHO reference values (22).

Pubertal stages were classified as follows: Tanner Stage 1 = Prepubertal stage; Tanner Stages 2–3 = Middle puberty; Tanner Stages 4–5 = Late puberty.

### 2.3 Metabolic and hormonal evaluation

Metabolic and hormonal profiles, such as total and HDL cholesterol, fasting plasma glucose (FPG), triglycerides (TG), free T3 (FT3), free T4 (FT4), and TSH, as well as fasting plasma insulin (FPI), were assessed at diagnosis within 24 h of admission and during follow-up. Blood samples were taken in a fasting state between 8:30 a.m. and 9:00 a.m.

The metabolic profile assessment encompassed TC, HDL-C, LDL-C, TG, insulin, glucose analyzed using the cobas® 6000 analyzer series (c501 and e601 module, Roche Diagnostics GmbH, Hoffmann-La Roche ltd, Mannheim, Germany).

Dyslipidemia was defined by TC > 200 mg/dl and/or HDL-C < 40 mg/dl and/or TG ≥ 100 mg/dl for ages 0–9 years; ≥130 mg/dl for ages 10–19 years) (23).

TG/HDL ratio, no-HDL/HDL ratio and atherogenic index of plasma (AIP), calculated as [log(triglycerides/HDL cholesterol)], was also considered as atherogenic risk markers. Specifically, a TG/HDL ratio <1.1, an LDL/HDL ratio <3, and an AIP <0.24 were defined as at-risk values (24–26).

Measurement of serum FT3, FT4 and TSH was obtained with the chemiluminescence immunoassay by Alinity/Abbott system (limit of detection: TSH 0.0083 μIU/ml; FT3 1.25 ng/dl; FT4 0.42 ng/dl) and the following normal ranges: FT3 3.5–6.3 pmol/L; FT4 9–19.3 pmol/L and TSH 0.5–4.2 μIU/ml. NTIS was defined as defined as any abnormal thyroid function result in the presence of critical illness and absence of a pre-existing abnormality in the hypothalamic-pituitary-thyroid axis. A thyroid ultrasound was performed on a patient with persistent alterations in TH levels during follow-up (Vscan Air™, GE Healthcare).

### 2.4 Statistical analysis

Categorical variables were expressed as counts and percentages; quantitative ones as median and interquartile range, as they were not normally distributed (normality was assessed using the Shapiro-Wilk test). The Wilcoxon test was used to compare hormonal and biochemical values at different points in time; a *p*-value of less than 0.05 was considered statistically significant. Spearman correlation analyses were conducted to explore potential relationships between thyroid and lipid values; the coefficient “*r*” was utilized for statistical correlation analyses. All statistical analyses were conducted using Stata software Stata software, version 17.0 (StataCorp, College Station, TX, USA).

## 3 Results

### 3.1 Hormonal and metabolic profile at admission

We included 56 patients (41M, 73.2% and 15M, 26.8%; mean age 8.6 ± 3.7 years). The details of the clinical characteristics at admission were described in Table 1.

**Table 1:** Patient characteristics at admission.

Characteristics	Value
Sex n. of patients (%)	
Males	41 (73.2)
Female	15 (26.8)
Age (years)	8.6 ± 3.7 years
BMI (kg/m^2^)	18.4 ± 3.0
BMI z-score	0.14 ± 1.10
Pubertal stage, n. of patients (%)	
Pre-pubertal	31 (55.3)
Middle puberty	16 (28.6)
Late puberty	9 (16.0)
Number of organs involved, n. of patients (%)	
2	21 (37.5)
3	17 (30.4)
4	18 (32.1)
Overall severity score	12.2 ± 3.2
Pediatric Intensive Care Unit admission, n. of patients (%)	38 (67.8)
Pediatric Intensive Care Unit stay (median days)	2
Non invasive ventilation, n. of patients (%)	22 (39.2)
Hospital stay (median days)	13

In Table 2, the thyroid hormones levels and lipid profile at admission are reported.

**Table 2:** Level of thyroid hormones and lipid profile. Data are expressed as median (IQR).

Parameters	Time at diagnosis	Time 6 months (T6)	Time 12 months (T12)	*p* (T0 vs. T6)	*p* (T0 vs. T12)
FT3 (nv 3.5–6.3)	2.5 (1.5)	5.55 (1.3)	5.3 (1.05)	<0.001	<0.001
FT4 (nv 9–19.3)	11.5 (3.3)	12.1 (1.45)	12.6 (2.0)	0.33	0.11
TSH (nv 0.5–4.2)	2 (1.88)	1.84 (0.95)	1.72 (1.01)	0.14	0.29
Tryglicerides (TG, nv < 100 mg/dl for ages 0–9 years; <130 mg/dl for ages 10–19 years)	164.5 (143.5)	70 (33.5)	53 (33.0)	<0.001	<0.001
Total cholesterol (TC, nv < 200)	116.5 (70)	150.5 (32)	151 (36)	<0.001	<0.001
HDL cholesterol (HDL, nv < 40)	16.5 (15)	47 (11.5)	48 (10.5)	<0.001	<0.001
TG/HDL (nv < 1.1)	9.96 (18.6)	1.51 (0.72)	1.71 (0.68)	<0.001	<0.001
No HDL/HDL (nv < 3)	6.89 (8.49)	2.13 (0.88)	1.98 (0.63)	<0.001	<0.001
Atherogenic index of plasma (nv < 0.24)	2.30 (1.37)	0.41 (0.48)	0.16 (0.62)	<0.001	<0.001
Fasting blood glucose	104.0 (38.0)	87.0 (8.00)	87.0 (8.00)	<0.001	<0.001
Fasting insulin	12.4 (11.10)	8.75 (9.85)	9.10 (9.70)	<0.001	<0.001
HOMA-IR	4.33 (2.67)	1.89 (2.30)	1.98 (2.29)	<0.001	<0.001

On admission, pathological levels FT3 (76.8%), FT4 (13.2%), TSH (14.5%) were detected (Table 3). All subjects present almost one pathological atherogenic risk markers. Specifically pathological TG was recorded in 46.4%, TC in 95.25%, HDL in 33.33%, TG/HDL ratio in 100%, no-HDL/HDL ratio in 91.67% and AIP in 100%. In 78.6% of the patients with pathological atherogenic risk markers, NTIS was also noted. In Table 3, patterns of hormonal and metabolic abnormalities were resumed.

**Table 3:** Patterns of hormonal and metabolic abnormalities.

Biochemical variables	0	6	12
Pathological FT3 (nv 3.5–6.3 pmol/L)	76.8%	19.6%	0%
Pathological FT4 (nv 9–19.3 pmol/L)	13.2%	1.79%	0%
Pathological TSH (nv 0.5–4.2 μIU/ml)	14.5%	7.15	0%
Pathological Tryglicerides (nv < 150 mg/dl)	46.4%	3.57%	3.57%
Pathological Total cholesterol (nv < 200 mg/dl)	95.25%	3.57%	0%
Pathological HDL cholesterol (nv > 40 mg/dl)	33.33%	19.64%	14.29%
Pathological Tryglicerides/HDL cholesterol (nv < 1.1)	100%	80.36%	56.57%
Pathological No HDLcholesterol/HDL cholesterol (nv < 3)	91.67%	16.07%	14.29%
Pathological Atherogenic index (nv < 0.24)	100%	67.86%	37.50%

Patients with both NTIS and pathological atherogenic risk markers showed a higher severity score compared to those with only NTIS (13.0 ± 3.1 vs. 11.2 ± 1.87, *p* = 0.05).

As reported in Table 4, correlation analyses revealed associations between FT3 and TG (*r* = 0.35), TC (*r* = 0.37) HDL (*r* = 0.38), TG/HDL (*r* = −0.26) No HDL/HDL (*r* = −0.33) and AIP (*r* = −0.27); between FT4 and TG (*r* = 0.24), TC(*r* = 0.36) HDL (*r* = 0.41), TG/HDL (*r* = −0.28) No HDL/HDL (*r* = −0.36) and AIP (*r* = −0.28) and between TSH and TG (*r* = 0.31) and TG/HDL (*r* = 0.33) and between TSH and TG (*r* = 0.25).

**Table 4:** Correlation between thyroid hormones and lipid markers and glycemic parameters. Data are expressed as Spearman'*s correlation* coefficient, *r*.

Metabolic parameter	FT3						FT4						TSH					
	T0		T6		T12		T0		T6		T12		T0		T6		T12	
	*r*	*p*	*r*	*p*	*r*	*p*	*r*	*p*	*r*	*p*	*r*	*p*	*r*	*p*	*r*	*p*	*r*	*p*
Tryglicerides	0.35	0.10	0.01	0.94	0.01	0.93	0.24	0.09	−0.15	0.26	−0.07	0.62	0.25	0.09	0.12	0.40	0.06	0.06
Total cholesterol	0.37	<0.01	0.26	0.05	−0.06	0.65	0.36	0.04	0.10	0.48	0.08	0.54	0.16	0.27	0.23	0.10	0.19	0.09
HDL cholesterol	0.38	0.02	0.06	0.65	−0.16	0.23	0.41	0.37	0.29	0.02	−0.02	0.86	0.03	0.84	−0.11	0.05	0.11	0.32
TG/HDL	−0.26	0.07	−0.01	0.93	0.04	0.78	−0.28	0.04	−0.21	0.37	−0.04	0.76	0.18	0.23	0.06	0.67	0.12	0.38
No HDL/HDL	−0.33	0.02	0.16	0.22	0.06	0.65	−0.36	0.01	−0.12	0.11	0.11	0.42	−0.01	0.86	0.08	0.59	0.01	0.93
Atherogenic index of plasma	−0.27	0.05	0.0	1.0	0.04	0.74	−0.28	0.05	−0.16	0.23	−0.08	0.55	0.11	0.23	0.06	0.67	0.03	0.83
Fasting blood glucose	−0.14	0.31	0.27	0.04	0.008	0.95	−0.12	0.39	0.05	0.69	−0.20	0.08	−0.08	0.54	−0.36	0.93	0.24	0.06
Fasting insulin	0.09	0.57	0.13	0.34	0.08	0.27	0.32	0.05	−0.10	0.89	−0.15	0.28	−0.07	0.65	0.39	0.42	0.14	0.29
HOMA-IR	−0.01	0.92	0.15	0.27	0.05	0.57	0.18	0.28	−0.11	0,75	−0.15	0.25	−0.09	0.57	0.25	0.43	0.14	0.31

T0 = at admission; T6 = 6-months follow-up; T12 = 12-months follow-up.

After a median hospital stay of 13 days, all patients made a full clinical recovery and were discharged.

### 3.2 Hormonal and metabolic assessment throughout follow-up

At the 6- and 12-month follow-ups, all patients demonstrated good general health, without recurrences.

Table 2 reports the hormonal and metabolic assessment over a 1-year follow-up period.

During follow-up, there was a significant improvement in FT3 levels, total and HDL cholesterol, TG, TG/HDL, No HDL/HDL, AIP, FPG, FPI (all *p* < 0.001). No ultrasound thyroid alteration was detected in patients with persistent alterations in TH levels.

Although complete restoration of TH balance was achieved at 12 months, some patients still exhibited an altered lipid profile (Table 3), without maintaining a correlation between FT3 and FT4 and lipid markers (all *r* < 0.2, Table 4). In particular, the pathological TG/HDL ratio persisted in 56.57% of patients, and AIP persisted in 37.50% of patients.

## 4 Discussion

The association between thyroid function and the lipid atherogenic profile in children with MIS-C has been a topic of investigation. Our findings indicate that MIS-C patients at diagnosis show a correlation between TH and biomarkers of dyslipidemia and atherogenic risk, underscoring the influence of TH on major metabolic pathways during the adaptive response to critical illnesses. During monitoring, an altered lipid profile persists even after the restoration of thyroid function, suggesting a possible predisposition to long-term metabolic complications. A role of the acute event as a trigger to unmask latent predisposed conditions cannot be excluded.

While children generally appear to be at lower risk for COVID-19, a small number may experience a rare but severe hyperinflammatory condition known as MIS-C (1–3, 27). As in our pediatric cohort, male predominance was noted (28, 29). No definite conclusions have been reached to explain the disparities between sexes. However, several hypotheses have been proposed to account for these differences, including the activity of X-linked genes, which modulate the innate and adaptive immune responses, as well as hormonal and genetic factors that influence the activity and expression of human angiotensin-converting enzyme 2 (ACE2), microRNA expression and transcription, and vitamin D3 activity (28).

Metabolic and endocrinological changes have been reported in children with MIS-C, particularly during the acute phase (1–3, 10, 11). Specifically, as also previously reported (10, 11), NTIS and dyslipidemia have been observed in the majority of children. The hyper-inflammatory state, cytokine storm, and adaptive metabolic response appear to play a significant role in these endo-metabolic changes (5, 6, 10, 11).

Proinflammatory cytokine activation are often linked to the lipid disorders. Research has shown that cytokines like TNF-α play a role in the severity of lipid disturbances. These cytokines are involved in various functions, including regulating energy balance, proliferation, and apoptosis of adipocytes, as well as lipolysis, inhibiting lipid synthesis, and reducing blood lipids (30).

Besides hyper-inflammatory condition, hypercatabolism, and metabolic adaptation, an imbalanced thyroid function plays a significant role in inducing alterations in the lipid metabolism (31, 32). THs affect synthesis, mobilization and degradation of lipids, although degradation is influenced more than synthesis (17).

Patients in critical illness, including infections, often experience significant physiological stress, which can affect multiple organ systems. In response to critical illness, the adaptive response allows vital organs to conserve energy, driven by counter-regulatory hormones and cytokines, leading to altered IR markers and lipids (33, 34). The metabolic changes following MIS-C are not dissimilar to other critical conditions. The persistence of the hypermetabolic response, surpasses the ability of the patient to respond, and physiological exhaustion ensues, leading to pathological metabolic changes (35). Such as alterations in the lipid and glucose profiles, as observed in our pediatric patients.

In the same way, as reported infections, sepsis, trauma and burns, several changes in TH may occur. Specifically, NTIS, has been reported during the COVID-19 pandemic, in adults during acute infection (8) and in children with MIS-C (10). NTIS is marked by a swift decline in serum triiodothyronine (T3) levels, which is why it is often referred to as “low T3 syndrome.” In NTIS, the reduction in T3 and thyroxine (T4) levels typically does not coincide with an increase in serum thyroid-stimulating hormone (TSH). On the contrary, serum TSH levels may decrease as a result of inhibition of the hypothalamic-pituitary-thyroid (HPT) axis. In critically ill patients NTIS is a metabolic adaption to stress (9) and it may be associated with poor outcomes (36). We observed that patients with both NTIS and pathological atherogenic risk markers exhibited increased disease severity. This finding supports the negative correlation between THs and metabolic markers, as well as their relationship with disease severity【 calcaterra, highlighting a plausible influence of an atherogenic profile on disease severity (15).

Considering the significant role of TH in regulating lipid metabolism (16, 17), our findings suggest that these changes may contribute to dyslipidemia and increased atherogenic risk markers upon admission, as indicated by the correlation between TH levels and an altered lipid profile (37).

Conversely, the persistence of an altered atherogenic lipid profile in some patients even after TH restoration may indicate that the changes in lipid profiles are not solely attributable to the levels of TH in these individuals. There could be a potential role of innate metabolic responses to viral infection and subsequent metabolic reprogramming that could occur (29). Furthermore, the interactions between adaptive responses and genetic predisposition to dyslipidemia should not be overlooked (38, 39) in children with MIS-C, particularly considering the potential link between COVID-19 and the exacerbation of hyperlipidemia in susceptible individuals. In fact, as noted by Schefelker et al. (39), during the COVID-19 pandemic, children with pre-existing dyslipidemia experienced worsening lipid profiles without accompanying weight gain.

The infection increases the risk of factors that may aggravate dyslipidemia, such as inflammatory responses, thyroid disorders, IR, and hyperglycemia (40). These factors are likely closely interconnected, as evidenced by our findings of correlations between THs and an altered atherogenic lipid profile, which are also observed in chronic conditions such as diabetes and metabolic syndrome (41–44).

The conditions of stress such as infectious episodes can be events that unmask individual predisposition to dyslipidemia. In our pediatric population upon admission, all patients presented with altered TG/HDL-C ratio and AIP, confirming that AIP levels are elevated in COVID-19 (15). These indices emerge as noteworthy markers in assessing cardiovascular risk, particularly for atherosclerosis; their persistent elevation during follow-up could constitute a risk factor in subsequent ages. Therefore, monitoring the metabolic profile during infections is important to identify early risk markers. In patients where a metabolic imbalance is observed during the acute phases of illness, it may be useful to reassess the metabolic profile over time and implement dietary and lifestyle adjustments early on as preventive strategies against cardiovascular events in adulthood.

We acknowledge several limitations in our study. First, the small sample size limits the robustness of our analysis, necessitating further multicenter studies to increase the sample size and validate our findings. Second, we were unable to compare metabolic and endocrinological data from the same patients before and during MIS-C to determine the direct impact of COVID-19 on these markers. Finally, we did not record any familial history of dyslipidemia to support the presence of a genetic predisposition.

In conclusion, our data support a relationship between thyroid function and atherogenic lipid profile in pediatric patients with MIS-C. This relationship may be due to an interaction between adaptive and innate metabolic responses and genetic predisposition. Considering that metabolic disorders and thyroid dysfunction increase the risk of severe disease due to viral infections, elucidating the intricate relationship between thyroid function and metabolic pathways during infections may help identify new biomarkers to prevent acute and fatal outcomes, thereby improving patient's prognosis. In clinical practice, monitoring the endocrine and metabolic profile may be useful for a better understanding of adaptive responses and for identifying patients with latent predisposed conditions. This at-risk group could gain from increased monitoring and more intensive management during these stressful events to improve outcomes and protect the child's long-term health.

## Data Availability

The original contributions presented in the study are included in the article, further inquiries can be directed to the corresponding author.
